# Single-cell RNA sequencing analysis identifies one subpopulation of endothelial cells that proliferates and another that undergoes the endothelial-mesenchymal transition in regenerating pig hearts

**DOI:** 10.3389/fbioe.2023.1257669

**Published:** 2024-01-15

**Authors:** Thanh Minh Nguyen, Xiaoxiao Geng, Yuhua Wei, Lei Ye, Daniel J. Garry, Jianyi Zhang

**Affiliations:** ^1^ Department of Biomedical Engineering, University of Alabama at Birmingham, Birmingham, AL, United States; ^2^ Department of Medicine, School of Medicine, University of Minnesota, Minneapolis, MN, United States; ^3^ Department of Medicine, Cardiovascular Diseases, University of Alabama at Birmingham, Birmingham, AL, United States

**Keywords:** single-nucleus RNA-sequencing, heart, angiogenesis, endothelial cells, cell cycle, autoencoder

## Abstract

**Background:** In our previous work, we demonstrated that when newborn pigs undergo apical resection (AR) on postnatal day 1 (P1), the animals’ hearts were completely recover from a myocardial infarction (MI) that occurs on postnatal day 28 (P28); single-nucleus RNA sequencing (snRNAseq) data suggested that this recovery was achieved by regeneration of pig cardiomyocyte subpopulations in response to MI. However, coronary vasculature also has a key role in promoting cardiac repair.

**Method:** Thus, in this report, we used autoencoder algorithms to analyze snRNAseq data from endothelial cells (ECs) in the hearts of the same animals.

**Main results:** Our results identified five EC clusters, three composed of vascular ECs (VEC1-3) and two containing lymphatic ECs (LEC1-2). Cells from VEC1 expressed elevated levels of each of five cell-cyclespecific markers (Aurora Kinase B [AURKB], Marker of Proliferation Ki-67 [MKI67], Inner Centromere Protein [INCENP], Survivin [BIRC5], and Borealin [CDCA8]), as well as a number of transcription factors that promote EC proliferation, while (VEC3 was enriched for genes that regulate intercellular junctions, participate in transforming growth factor β (TGFβ), bone morphogenic protein (BMP) signaling, and promote the endothelial mesenchymal transition (EndMT). The remaining VEC2 did not appear to participate directly in the angiogenic response to MI, but trajectory analyses indicated that it may serve as a reservoir for the generation of VEC1 and VEC3 ECs in response to MI. Notably, only the VEC3 cluster was more populous in regenerating (i.e., AR_P1_MI_P28_) than non-regenerating (i.e., MI_P28_) hearts during the 1-week period after MI induction, which suggests that further investigation of the VEC3 cluster could identify new targets for improving myocardial recovery after MI. Histological analysis of KI67 and EndMT marker PDGFRA demonstrated that while the expression of proliferation of endothelial cells was not significantly different, expression of EndMT markers was significantly higher among endothelial cells of AR_P1_MI_P28_ hearts compared to MI_P28_ hearts, which were consistent with snRNAseq analysis of clusters VEC1 and VEC3. Furthermore, upregulated secrete genes by VEC3 may promote cardiomyocyte proliferation via the Pi3k-Akt and ERBB signaling pathways, which directly contribute to cardiac muscle regeneration.

**Conclusion:** In regenerative heart, endothelial cells may express EndMT markers, and this process could contribute to regeneration via a endothelial-cardiomyocyte crosstalk that supports cardiomyocyte proliferation.

## 1 Introduction

Adult mammal hearts, especially the cardiomyocytes, cannot regenerate (Laflamme and Murry, 2011; Steinhauser and Lee, 2011); after being damaged by an acute injury, such as myocardial infarction (MI), a large amount of cardiomyocytes die (Chiong et al., 2011). Although the adult and juvenile hearts may undergo remodeling (Sutton and Sharpe, 2000) to attempt repairing the damage, cardiomyocytes undergo pathological hypertrophy instead of proliferation, leading to further decrease of heart function, left ventricle dilation, and heart failure (McMullen and Jennings, 2007). No approach to reverse the post-MI pathological remodeling has been found; therefore, treatments of MI are only temporary, such as strengthening the heartbeat and increasing blood flow to the heart (Lu et al., 2015); and heart failure still frequently occurs (McMullen and Jennings, 2007). On the other hand, 4 weeks after MI was induced in postnatal (P) day 1, mammal hearts recovered completely by P30 with no decline in cardiac function and negligible myocardial scarring (Porrello et al., 2011; Ye et al., 2018; Zhu et al., 2018; Zhao et al., 2020); in these cases, cardiomyocytes robustly proliferated instead of becoming hypertrophic. Therefore, studying how neonatal hearts undergo ‘regenerative’ remodeling instead of ‘failure’ remodeling is important to find better treatment for MI and heart failure. Furthermore, We demonstrated that when apical resection (AR) surgery is performed in pig hearts on P1 (AR_P1_), cardiomyocytes proliferated in response to MI induction on P28 (MI_P28_), and the animals fully recovered with no evidence of scar formation and also no decline in heart function by P56 (Zhao et al., 2020; Nakada et al., 2022). Thus, we established a model where the juvenile hearts perform a ‘regenerative’ remodel to repair MI damage. Although inducing AR_P1_ may not be a therapeutic solution to prevent heart failure in mammals, this ‘juvenile’ regenerative model is more mature than the neonatal P1; therefore, understanding how different the cardiac cell types’ regeneratively’ respond to MI in this model may lead to better approaches for treating MI in adult hearts. The single nuclei RNA sequencing data (snRNAseq), which encompassed the gene expression of not only cardiomyocyte but also other cardiac cell types, were generated from these AR_P1_MI_P28_ regenerative hearts, the age-match naïve hearts (including embryonic day 80, postnatal P1, P28, and P56), and age-match non-regenerative hearts (MI_P28_ only) (Nakada et al., 2022). The cardiomyocyte snRNAseq data were analyzed in (Nguyen et al., 2022; Nguyen et al., 2023a) via our Autoencoder, and ten cardiomyocyte subpopulations were identified, one of which was present only in cardiomyocytes from AR_P1_ hearts on P28 and appeared to proliferate in response to MI (Nguyen et al., 2022); the subpopulation upregulated T-box transcription factors 5, 20 (TBX5 and TBX20), and erb-b2 receptor tyrosine kinase 4 (ERBB4), which were known to promote mouse cardiomyocyte proliferation (Bersell et al., 2009; Maitra et al., 2009; Chakraborty and Yutzey, 2012; Chakraborty et al., 2013; Misra et al., 2014; Xiang et al., 2016).

On the other hand, how the endothelial cells responded to MI in regenerative hearts was poorly studied. The primary function of the coronary vasculature is to deliver oxygen and nutrients to myocardial cells while removing waste products. The transport and transfer of these materials are facilitated by the endothelium. Thus, regeneration of the vessels that are damaged by MI is essential for minimizing the loss of pre-existing cardiac cells and promoting cardiac repair (Khurana et al., 2005; Ingason et al., 2018; Singh et al., 2022). The endothelial cells (ECs) that line the coronary vasculature are highly specialized for each vessel category (arteries, veins, capillaries, and lymph vessels) and for different segments within each category (Becker et al., 2023). Even adjacent ECs within the same vessel can display substantial phenotypic differences, and this heterogeneity is further exacerbated by myocardial injury. For the experiments described in this report, we used the Autoencoder to analyze snRNAseq data from ECs in the same animal hearts, and because ECs in the coronary vasculature rarely proliferate in healthy hearts but are robustly proliferative in response to injury (Potente et al., 2011), we focused our analyses on the angiogenic response to MI by restricting our cluster analysis to only the 1,646 genes included under the ontology term “cell cycle” in the Gene Ontology Resource (Gene Ontology Annotations, 2022). Our main hypothesis is whether EC subpopulations that highly expressed cell-cycle markers appeared in the regenerative heart data. Understanding through which molecules the EC increased proliferating, thus improving angiogenesis, may lead to new approaches in enhancing the vascular functions following MI. Also, we examined the potential mechanism of how EC subpopulations may crosstalk with signaling pathways in cardiomyocytes and promote cardiomyocyte proliferation, which is another contribution to deciphering heart regeneration.

## 2 Results

### 2.1 Cell-cycle-specific AI autoencoding and cluster analysis of pig snRNAseq data identified five EC clusters

Pigs underwent AR_P1_, MI_P28_, AR_P1_MI_P28_, or neither surgical procedure (CTL). Subsequent experiments confirmed that pigs in the AR_P1_MI_P28_ group, but not MI_P28_ animals, completely regenerated the myocardial tissue that was damaged by MI (Zhao et al., 2020; Nakada et al., 2022). snRNAseq was performed with myocardial tissue collected from MI_P28_ and AR_P1_MI_P28_ animals on P30, P35, P42, and P56; from AR_P1_ and CTL animals on P28 and P56; from CTL animals on P1, and from fetal pigs.

**FIGURE 1:**
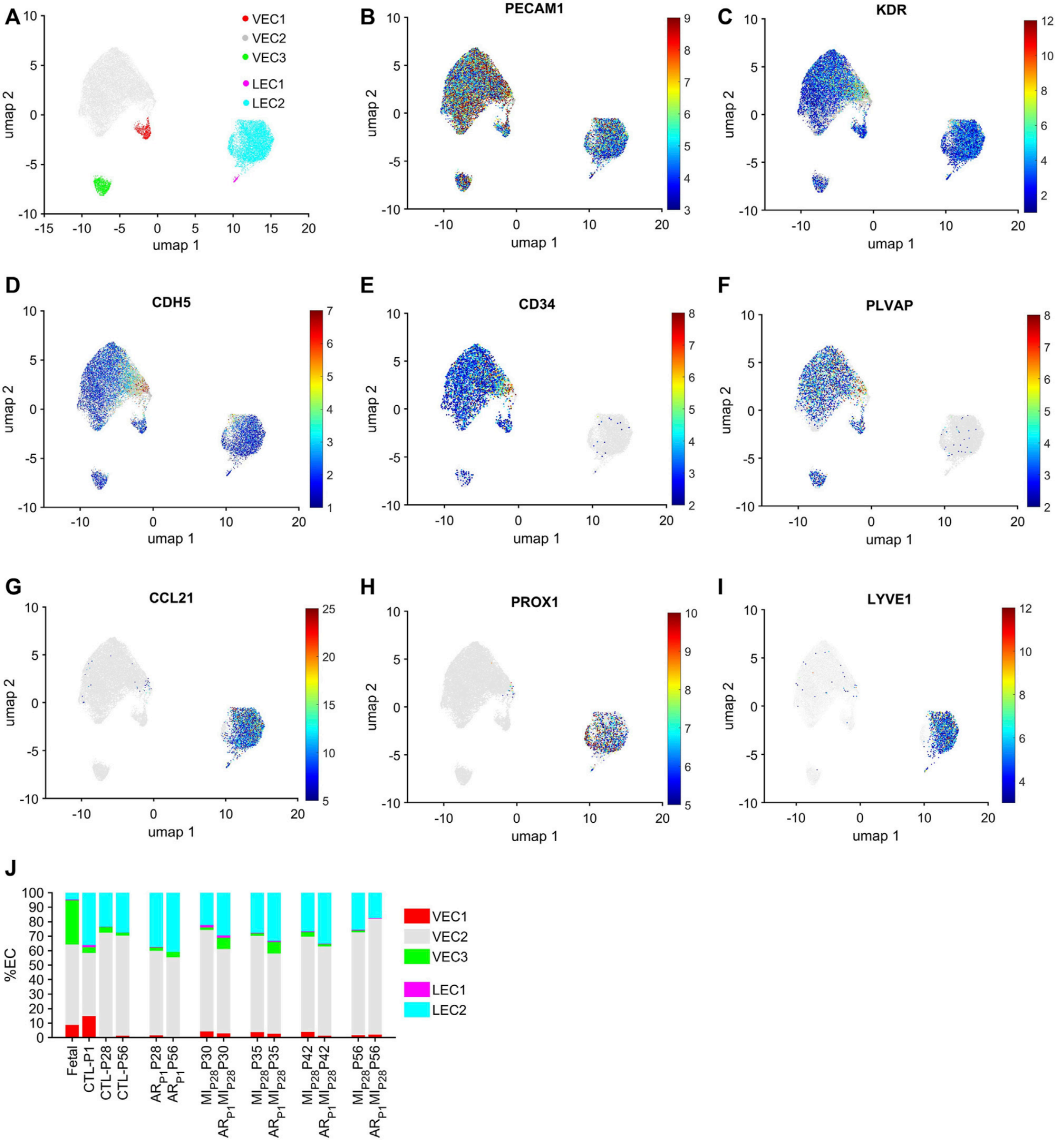
Cell-cycle specific Autoencoding defined five EC clusters in pig snRNAseq data. **(A)** Data for the expression of cell-cycle genes was extracted from the pig EC snRNAseq dataset, processed via an AI-based Autoencoder, and visualized in 2 dimensions (2D) via UMAP; the locations of each cluster are displayed. Cells that express the EC-specific markers **(B)** PECAM1, **(C)** KDR, and **(D)** CDH5; the VEC-specific markers **(E)** CD34 and **(F)** PLVAP; and the LEC-specific markers **(G)** CCL21, **(H)** PROX1 and **(I)** LYVE1 are displayed across the 2D cell-cycle–specific UMAP with the magnitude of expression represented as a color-coded heatmap; gray ECs did not express the corresponding molecule. **(J)** The proportion of ECs from each cluster is displayed for each injury group and time point.

The cell-cycle-specific Autoencoder (CSA) and clustering pipeline visualized five EC clusters (Figure 1A), each of which expressed elevated levels of the EC-specific surface markers Platelet And Endothelial Cell Adhesion Molecule 1 (PECAM1) (Newman, 1994), Kinase Insert Domain Receptor (KDR) (Terman et al., 1992), and vascular endothelial cadherin (CDH5) (Wu et al., 2021) (Figures 1B–D; Supplementary Figure S1). Three of the clusters were enriched for expression of the vascular-EC (VEC) markers CD34 (Fina et al., 1990) and Plasmalemma Vesicle Associated Protein (PLVAP) (Denzer et al., 2023) (Figures 1E, F), while the other two expressed high levels of the lymphatic-EC (LEC) markers C-C Motif Chemokine Ligand 21 (CCL21) (Johnson and Jackson, 2010), Prospero Homeobox 1 (PROX1) (Wigle et al., 2002), and Lymphatic Vessel Endothelial Hyaluronan Receptor 1 (LYVE1) (Banerji et al., 1999) (Figures 1G–I). Thus, the CSA appeared to separate the global cardiac EC population into three clusters of vascular ECs (VEC1-3) and two clusters of lymphatic ECs (LEC1-2).

Each EC cluster contained cells from multiple experimental groups and time points (Supplementary Figure S2), confirming that our results were not compromised by sampling bias. Further, we verified that the results were not compromised by batch effects. VEC1-3 collectively comprised >95% of ECs in fetal hearts and 59%–81% of ECs in hearts from all nonfetal animal groups (Figure 1J), whereas LEC1-2 included the remaining <5% of ECs in fetal hearts and 19%–41% of ECs in nonfetal hearts. The majority of VECs were clustered in VEC2, regardless of animal groups or time points, with the highest proportions of VEC1 and VEC3 ECs observed in CTL hearts on P1 and in fetal hearts, respectively. Nearly all LECs were clustered in LEC2.

### 2.2 VEC1 was enriched for the expression of genes involved in cell-cycle activity and proliferation

**FIGURE 2:**
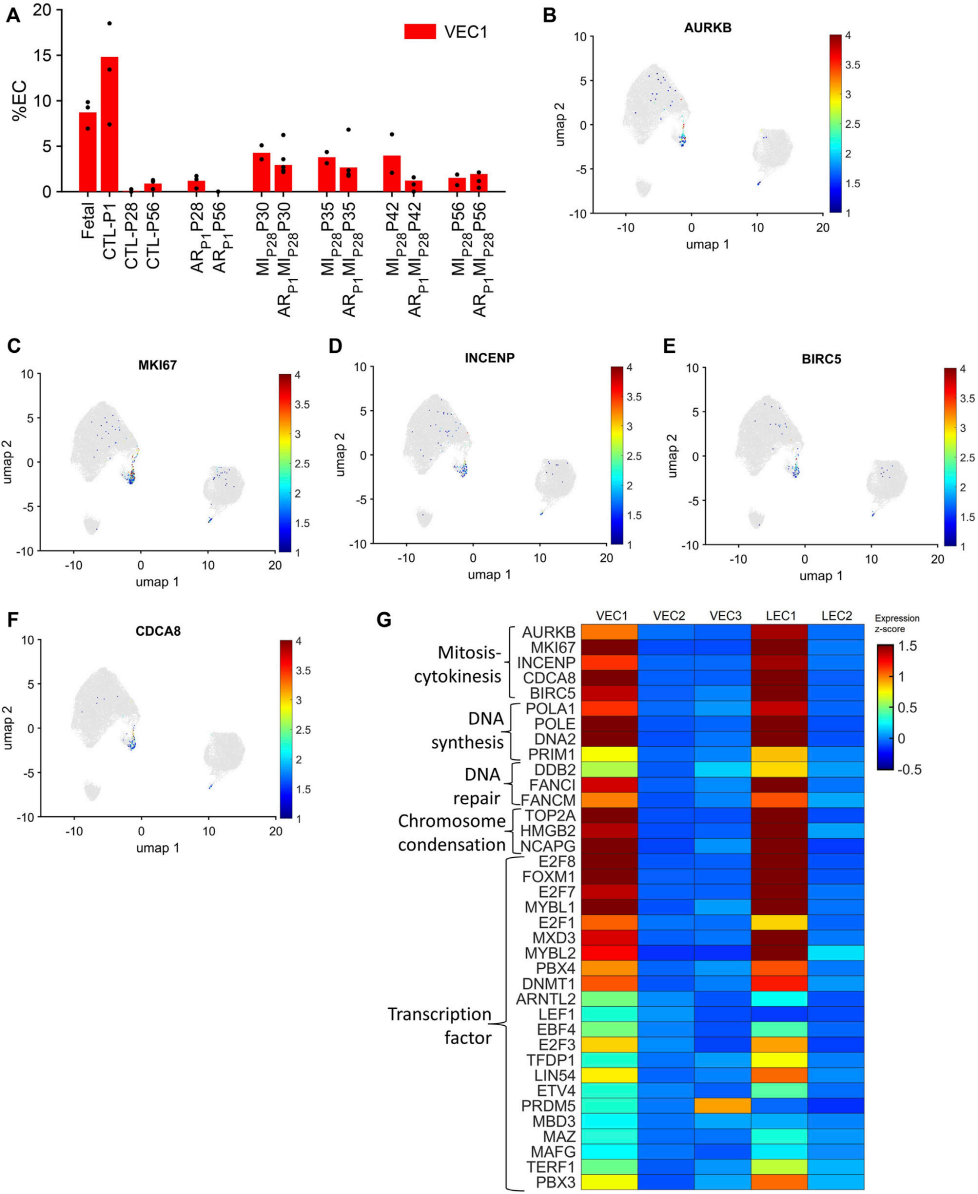
The VEC1 cluster is enriched for the expression of cell-cycle–related genes. **(A)** Data for VEC1 from Figure 1I is displayed with the *y*-axis rescaled for clarity. Cells that express the mitosis and cytokinesis markers **(B)** AURKB, **(C)** MKI67, **(D)** INCENP, **(E)** BIRC5, and **(F)** CDCA8 are displayed across the 2D cell-cycle–specific UMAP with the magnitude of expression represented as a color-coded heatmap; gray ECs did not express the corresponding molecule. **(G)** The abundance of expression for genes that are involved in mitosis and cytokinesis, DNA synthesis, DNA repair, and chromosome condensation, and for cell-cycle–regulating transcription factors is displayed for each cluster as a color-coded heat map (normalized by Seurat and converted to z-score).

VEC1 ECs comprised 8.7% of ECs in fetal hearts and 14.8% of ECs in CTL hearts on P1, they declined to nearly undetectable levels in CTL hearts on P28, and then expanded to include 3.5%–4.2% of ECs in MI_P28_ hearts from P30-P42 and in AR_P1_MI_P28_ hearts on P30-P35 (Figure 2A). VEC1 was also the only VEC cluster to express elevated levels of each of five markers for mitosis and cytokinesis (Aurora Kinase B [AURKB], marker of proliferation Ki-67 [MKI67], Inner Centromere Protein [INCENP], Survivin [BIRC5], and Borealin [CDCA8] (Li et al., 1998; Vader et al., 2006); Figures 2B–G; Supplementary Figure S3) and was enriched for the expression of genes associated with DNA synthesis and repair (Gene Ontology Annotations, 2023b), chromosome condensation (Gene Ontology Annotations, 2023c), and the gene ontology terms DNA replication (*p* = 3.86 × 10^−8^), mitotic spindle organization (*p* = 8.71 × 10^−7^), and mitotic cytokinesis (*p* = 2.42 × 10^−6^) (Figure 2G; Supplementary Tables S1A, B), as well as numerous transcription factors that have been linked to increases in EC proliferation and angiogenesis (Figure 2G; Table 1). Collectively, these observations are consistent with previous reports that the coronary vasculature undergoes a period of rapid growth and remodeling shortly after birth (Tian et al., 2014; Tan and Lewandowski, 2020) and suggest that a small proportion of cardiac ECs revert to a more proliferative phenotype in response to MI on P28.

**TABLE 1:** Summary of upregulated transcription factors in VEC1.

Transcription factor	Proportion of expressing cells (%)	Fold-change	*p*-value	References
E2F8	23.49	55.63	2.44 × 10^−157^	Weijts et al. (2012)
FOXM1	22.11	42.54	4.58 × 10^−132^	Zhao et al. (2006)
E2F7	15.89	27.14	1.57 × 10^−89^	Weijts et al. (2012)
MYBL1	29.88	17.78	1.28 × 10^−146^	Zhu et al. (2022)
E2F1	13.47	16.20	4.25 × 10^−63^	Qin et al. (2006)
MXD3	21.07	14.89	4.56 × 10^−89^	Diebold et al. (2019)
MYBL2	30.22	9.13	3.65 × 10^−102^	
PBX4	19.34	8.35	1.60 × 10^−55^	Martinou et al. (2022)
DNMT1	42.49	5.07	9.34 × 10^−96^	Zhang et al. (2016)
ARNTL2	13.99	3.84	4.37 × 10^−25^	
LEF1	10.19	3.09	1.59 × 10^−12^	Planutiene et al. (2011)
EBF4	20.55	3.08	8.00 × 10^−32^	
E2F3	51.47	3.08	6.77 × 10^−82^	Zhou et al. (2013)
TFDP1	11.40	2.92	2.75 × 10^−18^	
LIN54	49.91	2.87	3.05 × 10^−72^	
ETV4	11.74	2.76	2.51 × 10^−17^	Harel et al. (2021)
PRDM5	16.75	2.32	2.38 × 10^−18^	
MBD3	10.19	2.26	2.34 × 10^−13^	Yan et al. (2022)
MAZ	14.85	2.22	1.16 × 10^−16^	Smits et al. (2012)
MAFG	11.74	2.07	3.31 × 10^−13^	
TERF1	43.70	2.01	4.83 × 10^−37^	
PBX3	80.66	2.01	2.43 × 10^−62^	Chen et al. (2020), Wu et al. (2020)

Results are summarized as the proportion of VEC1 ECs that express the transcription factor, the fold-change in expression relative to expression in non-VEC1 ECs, and the *p*-value (Fisher’s Exact test).

**FIGURE 3:**
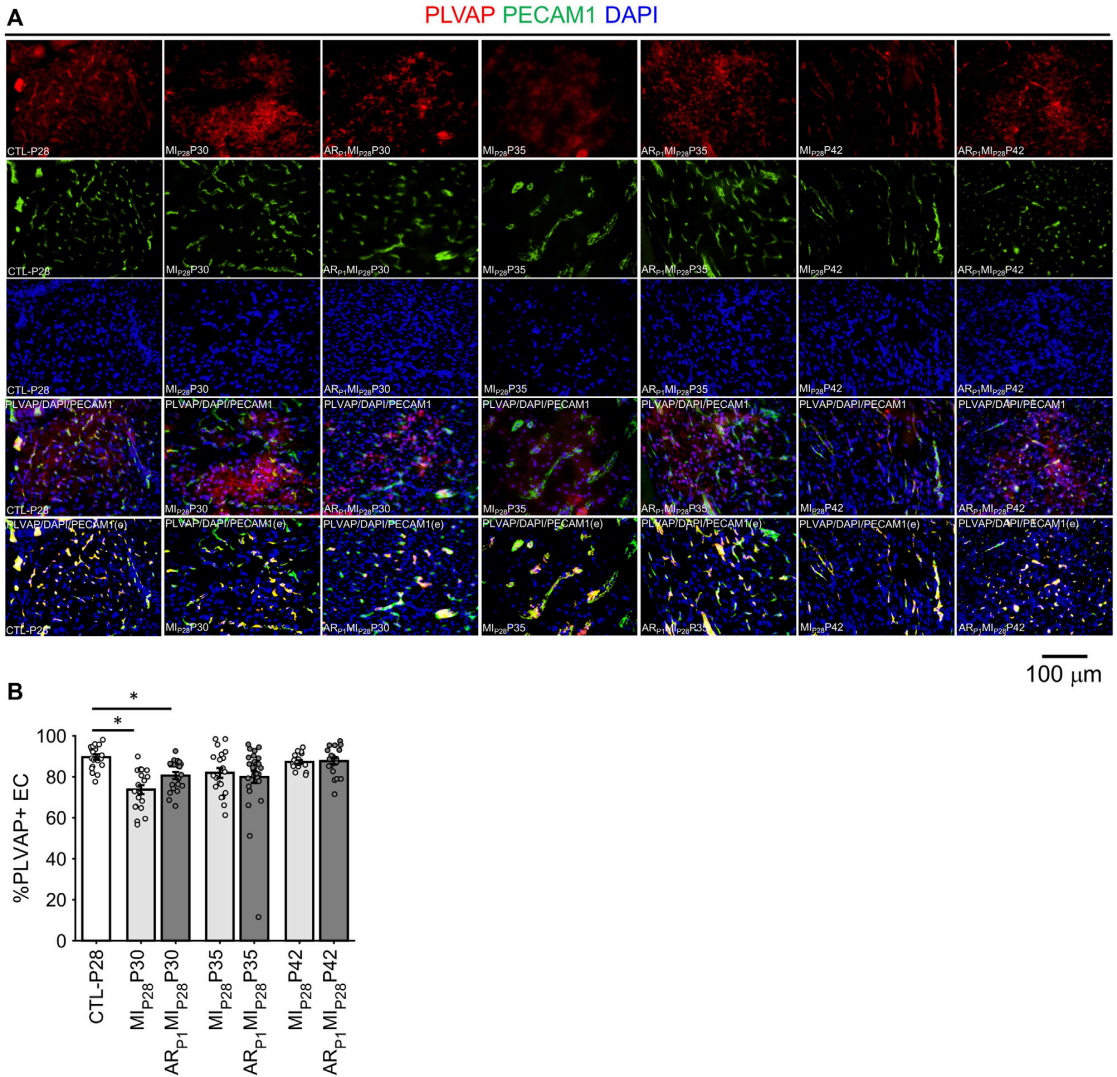
Fluorescence immunostaining analysis to assess ing the percentage of PLVAP + endothelial cells (VEC marker) in all animal group. **(A)** Representative images; the number of endothelial cells, marked by the DAPI blue fluorescence overlapping with EC marker PECAM1 green fluorescence, was counted; then, PLVAP red fluorescence and green fluorescence was amplified by 2x; red the signal not overlapping with green was removed before merging with DAPI (PLVAP/DAPI/PECAM(e) channel) to enhance PLVAP + EC visualization and counting. **(B)** Percentage of PLVAP + EC in all animal groups; the horizontal bar and the star (*) indicates non-parametric Wilcoxon’s ranksum test between two sample groups. *: *p*-value < 0.05.

Notably, although LEC1 ECs were exceptionally rare (<1.5%) in hearts from all groups and time points, they were most abundant in CTL hearts on P1 and in MI_P28_ and AR_P1_MI_P28_ hearts on P30 (Figure 1I; Supplementary Figure S4), and were enriched for many of the same mitosis/cytokinesis markers, gene ontology terms, and transcription factors that were upregulated in VEC1 ECs (Figure 2G), which suggests that expansion of the LEC1 cluster may have a role in the inflammatory response (Prabhu and Frangogiannis, 2016) and/or contribute to the re-uptake of interstitial fluid after MI. Also, Immunohistological staining of VEC clusters’ markers (PLVAP + PECAM1) (Figure 3) showed that on P30, the percentage of PLVAP + ECs significantly decreased, compared to CTL-P28 level as the baseline, following MI_P28_ in both regenerative (*p* = 3.382 × 10^−4^) and non-regenerative heart (*p* = 3.4995 × 10^−6^); then, these percentage returned to the baseline level on P42. Since the percentages of VEC and LEC clusters were complemented, this result also suggested an increase of LEC between 2 and 7 days after MI_P28_. This LEC expansion was also suggested by an increase of cycling LEC1 cluster found in the snRNAseq analysis. Besides, trajectory analysis of LEC clusters (Supplementary Figure S4) also showed continuous paths from LEC2 to LEC1; the ordered was from cell-cycle G1/S phase marker Cylin D1 (CCND1) through S-phase marker CCNE2, M-phase marker CCNB2, to proliferation marker MKI67.

### 2.3 VEC3 was enriched for the expression of genes involved in cell adhesion, the regulation of intercellular junctions, and the endothelial-mesenchymal transition (EndMT)

**FIGURE 4:**
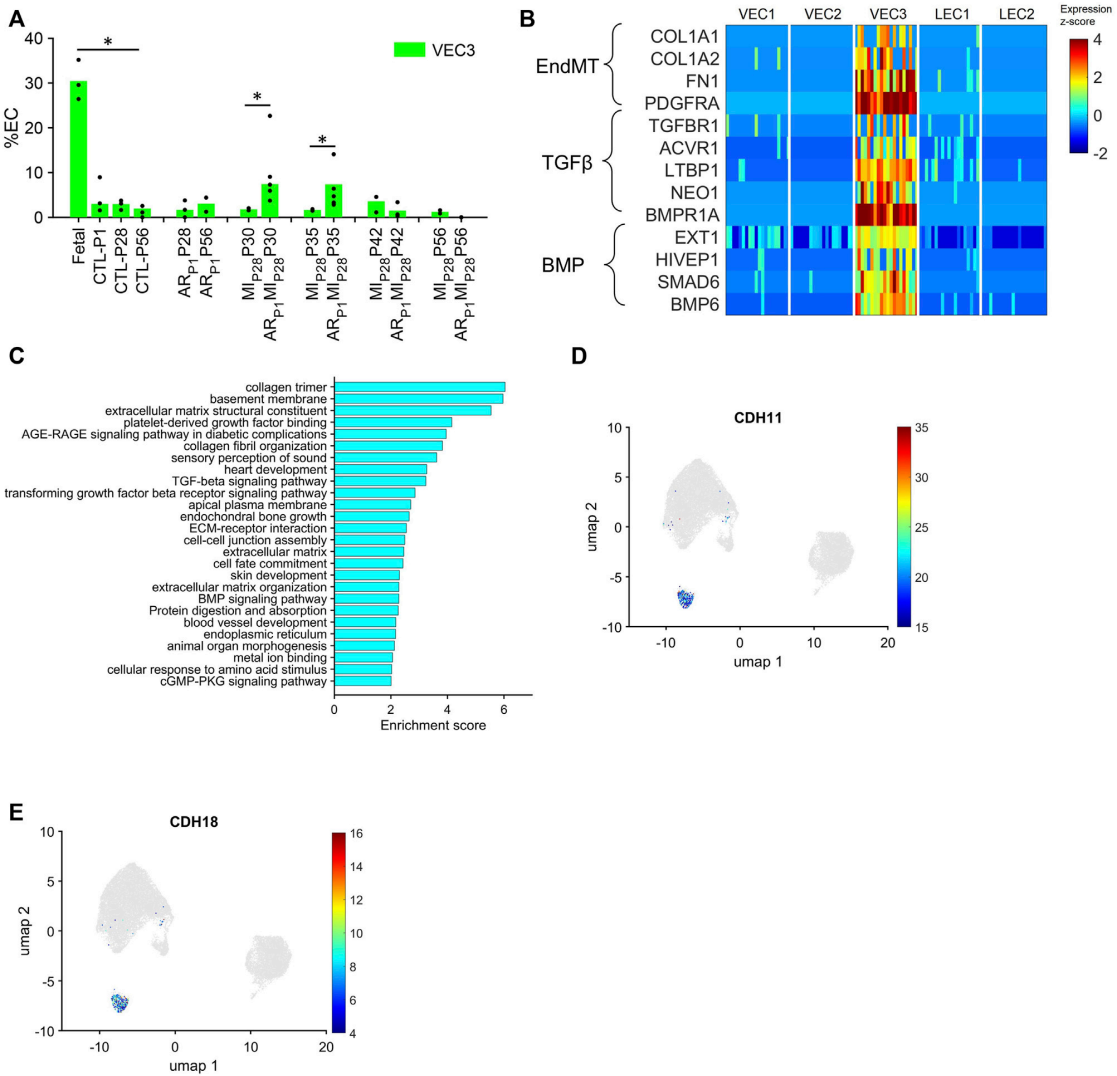
The VEC3 cluster is enriched for the expression of genes involved in the EndMT and cell adhesion. **(A)** Data for VEC3 from Figure 1I is displayed with the *y*-axis rescaled for clarity (*p < 0.05, Wilcoxon-ranksum test). **(B)** The abundance of expression for genes that are involved in the EndMT, in Transforming Growth Factor β signaling, and in Bone Morphogenetic Protein signaling (BMP) is displayed for each cluster as a color-coded heat map (normalized by Seurat and converted to z-score). **(C)** Enrichment scores (base-10 logarithm of enrichment *p*-value) were calculated for ECs from the VEC3 cluster and evaluated with the DAVID functional analysis tool; the 26 most highly enriched ontologies/pathways (*p* < 0.01) are displayed. **(D,E)** Cells that express the adhesion molecules **(D)** CDH11 and **(E)** CDH18 are displayed across the 2D cell-cycle–specific UMAP with the magnitude of expression represented as a color-coded heatmap; gray ECs did not express the corresponding molecule.

Although VEC1 ECs became more common in pig hearts after MI was induced on P28, they tended to be *less* common (though not significantly) in AR_P1_MI_P28_ hearts than in MI_P28_ hearts from P30-P42 and, consequently, are unlikely to contribute to the enhanced regenerative potential observed in the hearts of AR_P1_MI_P28_ pigs. Conversely, the VEC3 cluster was significantly *more* prominent in fetal (30.46%) than CTL hearts (3.9%–2.0% from P1-P56; *p* < 0.05 at each time point) and in AR_P1_MI_P28_ hearts than in MI_P28_ hearts on P30 (AR_P1_MI_P28_: 6.33%, MI_P28_: 1.6%; *p* < 0.05) and P35 (AR_P1_MI_P28_: 6.86%, MI_P28_: 1.8%; *p* < 0.05) (Figure 4A), but was *not* enriched for the expression of mitosis/cytokinesis markers (Figure 2G; Supplementary Table S2A). Instead, VEC3 ECs expressed elevated levels of genes associated with the EndMT, with the Transforming Growth Factor β (TGFβ) and Bone Morphogenetic Protein (BMP) (Figure 4B; Supplementary Table S2B) pathways (Huang et al., 2022; Gene Ontology Annotations, 2023a; KEGG, 2023), and with ontology terms that are associated with the upregulation of blood-vessel development (Figure 4C; Supplementary Table S2B). Notably, both TGFβ and BMP signaling regulate the EndMT, and although the EndMT appears to contribute to the formation of a number of pathological vascular abnormalities (Huang et al., 2022), all three processes are crucial for embryonic cardiovascular development (Gordon and Blobe, 2008; Morrell et al., 2016; Kovacic et al., 2019). Furthermore, VEC3 ECs expressed elevated levels of Plakophilin 2 (PKP2, fold-change: 24.62, *p*-value: 5.74 × 10^−247^), which regulates the assembly of intercellular junctions (Bass-Zubek et al., 2008), as well as the adhesion molecules Cadherin 11 (CDH11) and Cadherin 18 (CDH18) (Figures 4D, E; Supplementary Figure S5), while expression of the junctional proteins KDR and CDH5 (Giannotta et al., 2013) was somewhat lower in VEC3 than in VEC1 ECs (Supplementary Figure S1).

### 2.4 Trajectory analysis linked VEC2 to VEC1 and VEC3, but there was no direct pathway between VEC1 and VEC3

**FIGURE 5:**
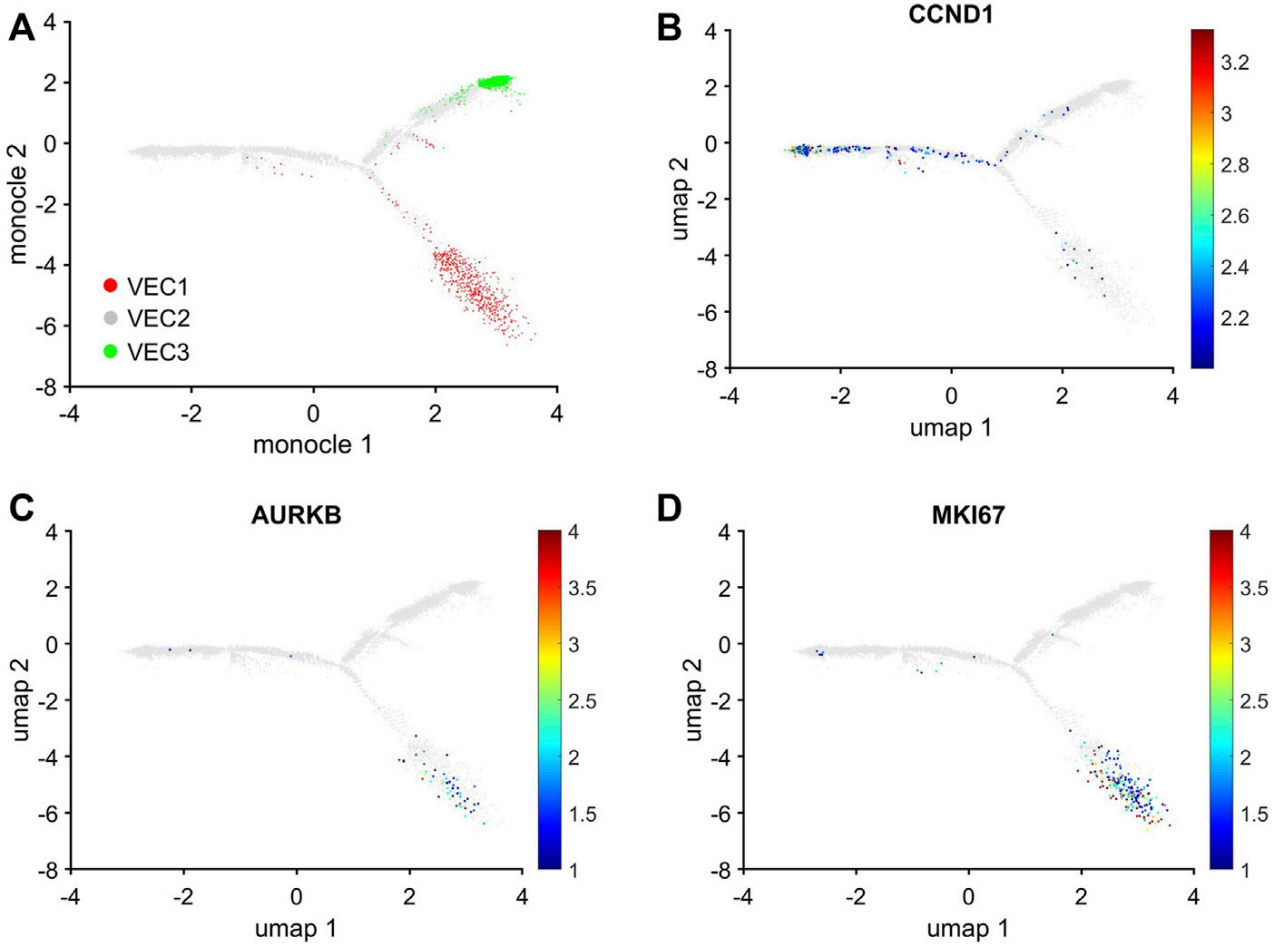
Trajectory analysis of pig EC snRNAseq data identified pathways between VEC2 and VEC1 or VEC3, but not between VEC1 and VEC3. Trajectory analysis of cell-cycle gene expression in VEC1, VEC2, and VEC3 ECs was performed via Monocle. **(A)** The positions of cells from each of the three VEC clusters is displayed across the trajectory. ECs that express **(B)** CCND1, which regulates the G1/S phase transition, and the proliferation markers **(C)** AURKB and **(D)** MKI67 are displayed across the 2D cell-cycle–specific UMAP with the magnitude of expression represented as a color-coded heatmap; gray ECs did not express the corresponding molecule.

The uniqueness of the VEC1 and VEC3 clusters was also evidenced by the results from Monocle pseudotime trajectory analysis (Qiu et al., 2017) of cell-cycle snRNAseq data for cells in all three VEC clusters. The analysis produced a branched structure with the tips of two branches each composed almost exclusively of VEC1 or VEC3 ECs and the third branch, as well as the region where all three branches converged, occupied by primarily VEC2 ECs (Figure 5A). Furthermore, the distribution of cells expressing Cyclin D1 (CCND1), which regulates the G1/S phase transition, and the proliferation markers AURKB and MKI67 traced a pathway between VEC2 and VEC1, but not between VEC2 and VEC3 or VEC3 and VEC1 (Figures 5B–D). Collectively, these observations indicated that VEC2 ECs could transform into either VEC1 or VEC3 ECs, but there was no direct transformational pathway between the VEC1 and VEC3 clusters, and identified a trajectory from VEC2 to VEC1 that recapitulated the onset and progression of cell-cycle activity and proliferation.

### 2.5 Angiogenesis (vessel density and proliferation marker)

**FIGURE 6:**
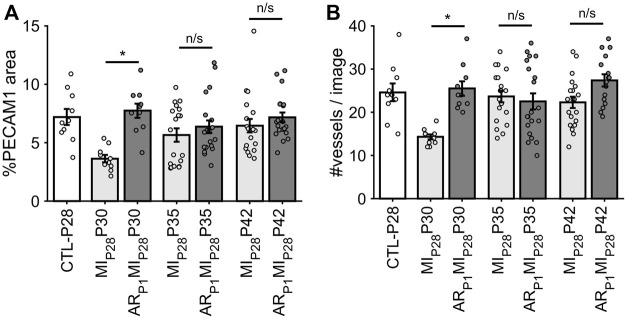
Estimating vessel density in each animal group via fluorescence immunostaining. **(A)** For each image, the percentage of pixel having PECAM1 green fluorescence was counted. **(B)** For each image, the number of vessels was manually counted. The horizontal bar and the star (*) indicates non-parametric Wilcoxon’s ranksum test between two sample groups. *: *p*-value <0.05; ns: *p*-value ≥ 0.05.

**FIGURE 7:**
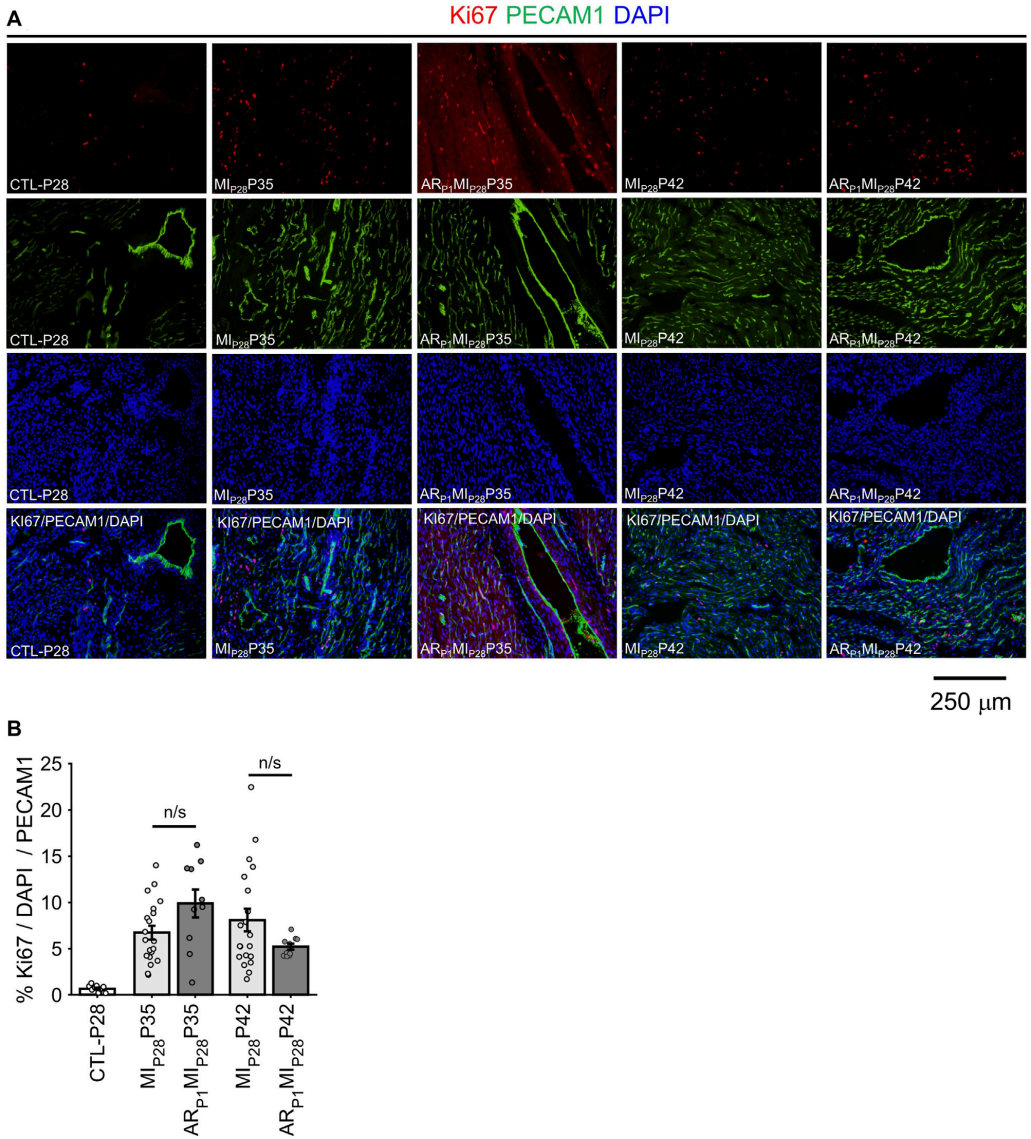
Immunofluorescence analysis confirming EC proliferation. **(A)** Representative images of Ki67 (red)/PECAM1 (green)/DAPI (blue) in each group. **(B)** For each image, the percentage of KI67+ EC was manually counted. The horizontal bar and the star (*) indicates non-parametric Wilcoxon’s ranksum test between two sample groups. ns: *p*-value ≥ 0.05.

Following MI_P28_, the vessel density, quantified by the percentage of image PECAM1 area and by the manual counting of vessels in each image (Figure 6), significantly decreased on P30 in non-regenerative hearts; then, on P35 and P42, their vessel densities recovered to the level of CTL-P28 hearts. Meanwhile, in regenerative hearts, the vessel densities did not significantly change, remaining similar to CTL-P28. On the other hand, Marker of Proliferation Ki67 immunofluorescence analysis (Figure 7) showed that seven to 14 days following the MI_P28_ injury, endothelial cells in both regenerative and non-regenerative hearts actively proliferate. Before the injury, less than 1% of EC expressed Ki67. Then, on day 7 and 14 after MI_P28_, the percentages of Ki67+ EC increased to, and remained between 6% and 8% in the non-regenerative hearts (MI_P28_P35 and MI_P28_P42, respectively); meanwhile, in the regenerative hearts, % Ki67+ EC increased to 9.88% on P35, then decreased to 5.20% on P42. The difference of Ki67+ EC between the two groups was insignificant at these time points (*p* = 0.062 and *p* = 0.467, respectively), and was consistent with the snRNAseq analysis. Then, we compared the expression of Vascular Endothelial Growth Factor A (VEGFA) and Vascular Endothelial Growth Factor B (VEGFB), which are known as the driver genes in angiogenesis involved in wound healing (Johnson and Wilgus, 2014), in the whole-heart snRNAseq data. Supplementary Figure S6 showed that it was unclear whether these two genes expressions were consistently higher in the regenerative (or on-regenerative) groups. This observation may explain why we did not observe significant angiogenesis between the regenerative and non-regenerative hearts.

### 2.6 VEC3 markers PDGFRA are highly expressed in ECs of regenerative hearts up to 7 days following MI_P28_ injury

**FIGURE 8:**
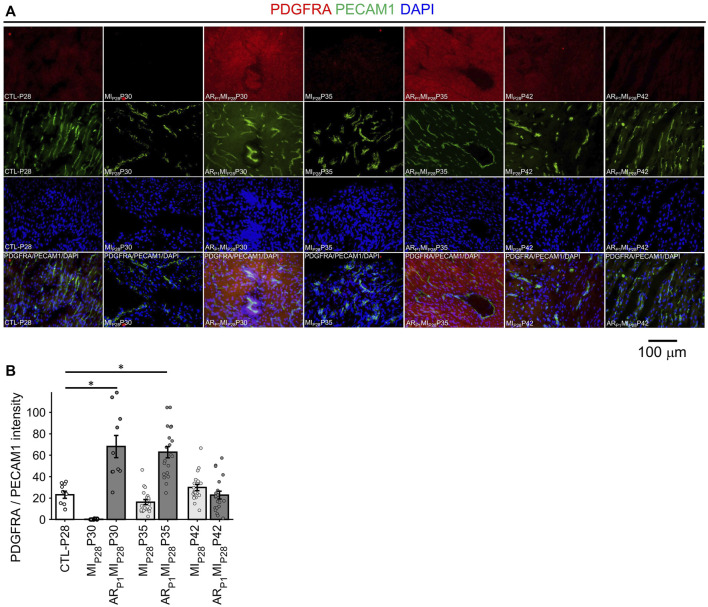
Fluorescence immunostaining analysis to confirm PDGFRA (EndMT marker) expression in endothelial cells. **(A)** Representative images of PDGFRA (red)/PECAM1 (green)/DAPI (blue) in each group. **(B)** The expression of PDGFRA was represented by the average PDGFRA red light intensity that are overlapped with EC marker green regions. The horizontal bar and the star (*) indicates non-parametric Wilcoxon’s ranksum test between two sample groups. *: *p*-value < 0.05.

In Figure 8, the expression of EndMT marker PDGFRA in EC was represented by the intensity of red fluorescence overlapping with green (EC marker PECAM1) fluorescence. A small number (∼1.5%) of EC in the CTL-P28 hearts were VEC3; therefore, the PDGFRA expression in CTL-P28 EC was used as the baseline. Then, following MI_P28_ injury, PDGFRA expression in the non-regenerative hearts EC sharply decreased on P30, then gradually increased on P35 and P42; however, the increased expression did not reach beyond the baseline level. Meanwhile, PDGFRA expression sharply increased in the regenerative heart on P30 (*p* = 7.69 × 10^−4^) and P35 (*p* = 4.71 × 10^−5^), then reverted to the baseline level on P42. These trends were consistent with the snRNAseq analysis. Also, surprisingly, PDGFRA immunofluorescence analysis showed that non-endothelial cells surrounding PDGFRA + EC also expressed PDGFRA; these cells could be cardiac fibroblasts and cardiomyocytes, as reported in (Bloomekatz et al., 2017; Yao et al., 2022).

### 2.7 VEC3 upregulated genes encoding secretable proteins that may promote cardiomyocyte proliferation and heart regeneration via Pi3k-Akt, Hippo/YAP, and ERBB4 signaling pathways

**FIGURE 9:**
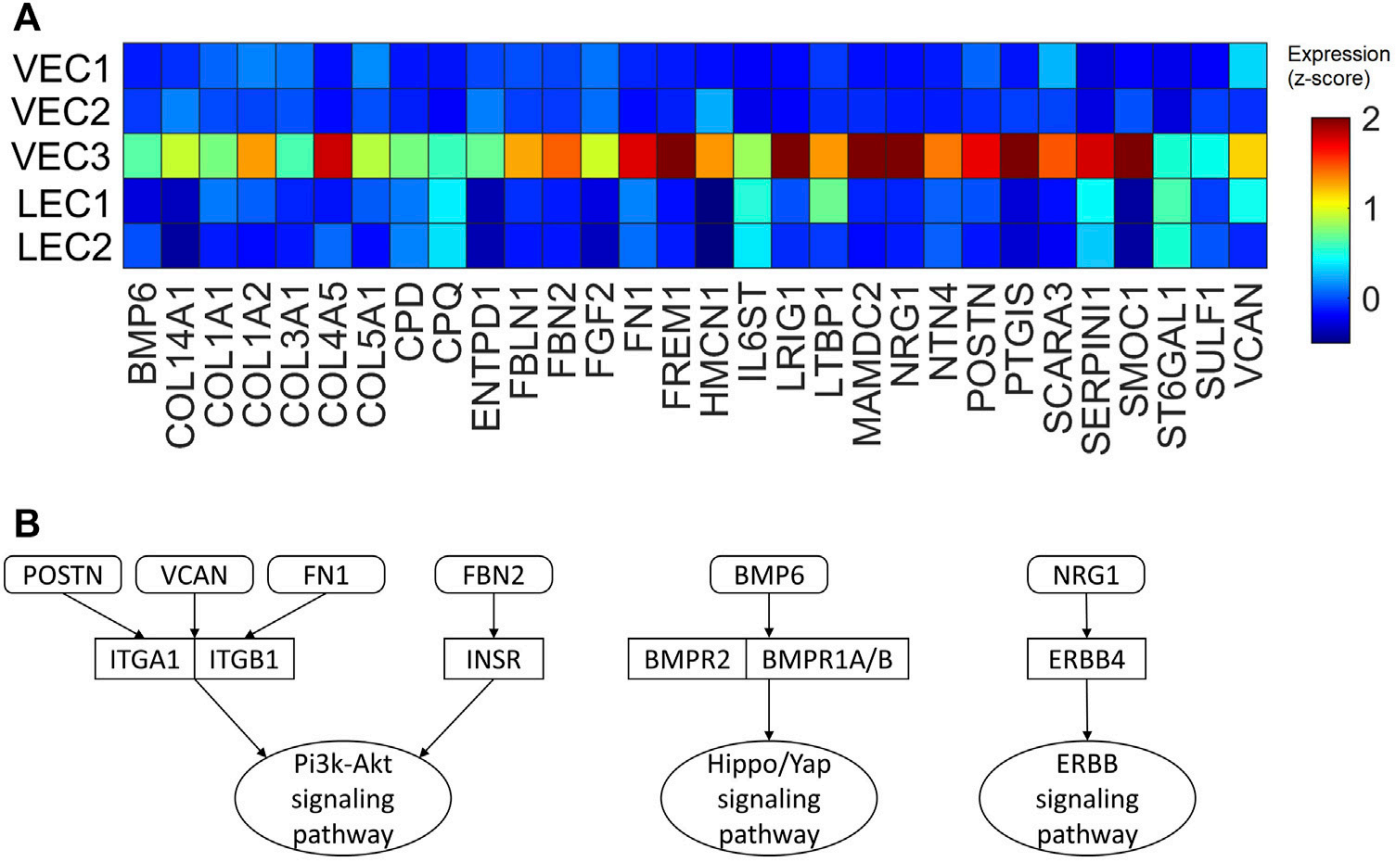
Upregulated genes encoding secretable proteins in cluster VEC3 interact with receptors of signaling pathways enriched in the regenerative hearts. **(A)** Heatmap of 30 genes encoding secretable proteins that exclusively upregulated (fold-change >2) in cluster VEC3; here, the expression is scaled by z-score and reflected in the heatmap color. **(B)** Protein-protein interactions, which were queried from BioGrid database, between the genes and their receptors of Pi3k-Akt, Hippo/YAP, and ERRB4 signaling pathways; round rectangle: genes; rectangle: signaling pathway receptors; oval: downstream signaling pathway.

Our previous works demonstrated the upregulation of Hippo/YAP signaling pathway in regenerative pig hearts that underwent myocardial infarction on P1 (Zhang et al., 2020), as well as ERBB4 in AR_P1_P28 hearts (Nguyen et al., 2022) at the protein level. Also, Pi3k-Akt signaling pathway was found enriched in AR_P1_MI_P28_ cardiomyocytes (Nakada et al., 2022; Nguyen et al., 2023b). Therefore, we examined whether the VEC3 cluster may have genes that interact with these pathways (Figure 9A). Thirty genes encoding secretable proteins were found upregulated in VEC3; among them, Fibronectin 1 [FN1] (Wang et al., 2013), Neuregulin 1 [NRG1] (Bersell et al., 2009; Gemberling et al., 2015), Versican [VCAM] (Feng et al., 2023), and Periostin [POSTN] (Kuhn et al., 2007) were known to promote cardiomyocyte proliferation in zebrafish, mice, and rat. We also queried the protein-protein interactions between these 30 genes and their receptors linked to Pi3k-Akt, Hippo/YAP, and ERBB4 signaling pathways in the Biological General Repository for Interaction Datasets (BioGrid) (Oughtred et al., 2021). Figure 9B shows that POSTN, FN1, and VCAM interact with Integrin Alpha 1 [ITGA1] and Integrin Beta 1 [ITGB1], which are receptors of the Pi3k-Akt signaling pathway (Feng et al., 2023). Also, Fibrillin 2 [FBN2], which was upregulated in VEC3, interact with Insulin Receptor [INSR] of Pi3k-Akt signaling pathway. Furthermore, NRG1 interacts with ERBB4, and Bone Morphogenetic Protein 6 [BMP6] interacts with receptors Bone Morphogenetic Protein Receptor Type 1A/B and Type 2 [BMPR1A/B, BMPR2] of the Hippo/Yap signaling pathway. Overall, these secret protein-receptor interactions suggested that VEC3 cluster may promote cardiomyocyte proliferation and overall regeneration.

## 3 Discussion

ECs comprise an exceptionally heterogeneous population of cells with unique features and functions that are specialized for each organ, for each vascular category (i.e., arteries, veins, capillaries, lymph vessels) within an organ, and for each segment within a vessel (Becker et al., 2023). The rate of EC turnover is believed to be low in most healthy organs, but the cells rapidly proliferate in response to injury (Potente et al., 2011). Thus, as we sought to characterize how different subpopulations of ECs participate in the angiogenic response to MI, we analyzed snRNAseq data from ECs that were collected during previous studies in our neonatal pig cardiac double-injury (AR_P1_MI_P28_) model via our cell-cycle–specific AI Autoencoder. Our analysis distributed the cells into five EC clusters, three containing VECs and two containing LECs.

Of the three VEC clusters, only one (VEC1) was enriched for cell-cycle activity, while another (VEC3) highly expressed genes involved in cell adhesion, in the regulation of intercellular junctions, in the EndMT, and in TGFβ/BMP signaling, which regulates the EndMT (Piera-Velazquez and Jimenez, 2019). Notably, angiogenesis is initiated by vessel sprouting (Sun et al., 2016), which begins with the loss of junctions between ECs and may be accompanied by onset of the EndMT in tip cells (Gerhardt et al., 2003); then, ECs located in the stalk of the sprout proliferate to lengthen and expand the endothelial tube (Marcelo et al., 2013). Thus, our observations suggest that VEC3 ECs may function as EC tip cells, while the VEC1 cluster could be composed primarily of stalk ECs. Furthermore, while ECs of the VEC2 cluster did not appear to be directly involved in angiogenesis, our trajectory analyses identified pathways linking VEC2 to both VEC1 and VEC3, which suggests that the VEC2 cluster contains a reservoir of ECs that can transform into tip (VEC3) or stalk (VEC1) ECs during angiogenesis. This observation is consistent with reports (Tian et al., 2015) that proliferating ECs are generated primarily from pre-existing ECs rather than another source, but we did not find a trajectory between VEC1 and VEC3, so the onset of EC cell-cycle activity does not appear to be driven by the EndMT. Notably, LEC1 ECs were enriched for many of the same mitosis/cytokinesis markers, gene ontology terms, and transcription factors that were upregulated in VEC1 ECs, which suggests that they also proliferate in response to MI and, consequently, may limit cardiac edema by increasing the uptake of interstitial fluid, but their precise role during recovery from myocardial injury has yet to be investigated.

The EndMT has been linked to increases in fibrosis (Fan et al., 2023), and recent studies in mice suggest that the acquisition of a mesenchymal-like phenotype in ECs after MI can lead to vascular abnormalities in newly formed vessels that block the efficient delivery of oxygen and other nutrients to the myocardium (Huang et al., 2022). These reports may appear to conflict with our observation that VEC3 ECs, which expressed elevated levels of EndMT genes, were the only EC subpopulation that was enriched in regenerating hearts. However, this discrepancy may be at least partially explained by differences in the prevalence and/or persistence of the mesenchymal-like phenotype: whereas the VEC3 cluster never contained more than ∼7.5% of ECs in nonfetal pig hearts and was expanded for just 1 week after MI induction in AR_P1_MI_P28_ pigs, the proportion of ECs that expressed mesenchymal genes in infarcted mouse hearts (Huang et al., 2022) increased from 1%–3% to 20%–70% during the first week after MI induction, and 5%–20% of them continued to express at least some mesenchymal genes for 3–8 weeks afterward. On the other hand, EC acquiring the EndMT markers may enhance their ability to proliferate, migrate, and dissociate (Huang et al., 2022). Our vessel density analysis showed that 2 days following MI_P28_ injury, the regenerative heart vessel density, where the VEC3 cluster strongly presented, quickly reached the CTL-P28 level; meanwhile, the non-regenerative heart, with a very small amount of VEC3, vessel density did not. VEC3 may promote cardiomyocyte proliferation and overall heart generation by secreting pro-proliferative proteins.

In conclusion, our analyses of cardiac snRNAseq data collected from fetal pigs and pigs that underwent AR_P1_, MI_P28_, both AR_P1_ and MI_P28_, or neither experimental injury identified three clusters of VECs and two LEC clusters. Cells from just one of the VEC clusters (VEC1) appeared to proliferate in response to MI induction, and a second VEC cluster (VEC3) was enriched for the expression of genes that regulate intercellular junctions, participate in TGFβ/BMP signaling and promote the EndMT, while the remaining VEC cluster (VEC2) appeared to provide a reservoir of ECs that transformed into VEC1 or VEC3 ECs after MI. Notably, although the roles of TGFβ/BMP signaling and the EndMT have been well-studied in cardiac development, their involvement in cardiac regeneration is not well understood. Thus, more in-depth studies of the VEC3 cluster could identify new targets for improving the regeneration of infarcted hearts.

## 4 Methods

### 4.1 snRNAseq datasets

The pig snRNAseq dataset was obtained from the Gene Expression Omnibus database (accession number GSE185289). The complete dataset included 250,700 cells and was processed as described previously (Nakada et al., 2022; Nguyen et al., 2022; Nguyen et al., 2023b). The number of pig hearts for each group was summarized in Supplementary Table S3. The data were normalized with Seurat v.4 (Hao et al., 2021); then, expression data for 14,753 genes with at least 1,000 unique molecular identifiers were embedded into 10 dimensions with an Autoencoder. The results were visualized via Uniform Manifold Approximation (UMAP), and clustering was performed with the UMAP density-based clustering (dbscan) algorithm (McInnes et al., 2018; Meehan, 2021). Cells in clusters that specifically expressed the EC markers PECAM1 and KDR were considered ECs. A total of 29,826 ECs were identified.

### 4.2 Animal heart tissues

Frozen tissues, from which the snRNAseq datasets (Nakada et al., 2022; Nguyen et al., 2022; Nguyen et al., 2023b) were generated, were cut into 1 cm thick sections, embedded in optimal cutting temperature compound, and preserved in −80°C freezers. All experimental protocols were approved by The Institutional Animal Care and Use Committee (IACUC) of the University of Alabama, Birmingham, and performed in accordance with the National Institutes of Health Guide for the Care and Use of Laboratory Animals (NIH publication No 85-23).

### 4.3 Cell-cycle-specific analyses

#### 4.3.1 Autoencoder and clustering

EC data was extracted from the full pig dataset, and then snRNAseq data for the 1,646 genes associated with the Gene Ontology term “cell cycle” (GO:0007049) (Gene Ontology Annotations, 2022) were reprocessed via AI Autoencoding and cluster analysis. This cell-cycle-specific Autoencoder was composed of three layers: an input layer of 1,646 nodes (i.e., the snRNA expression data for each of the 1,646 cell-cycle genes), a hidden (embedded) layer of 10 nodes, and an output layer of 1,646 nodes. The input layer was alternately embedded into the hidden layer and expanded to form the output layer, and the similarity of the input and output layers was optimized by minimizing the following equation:

$$E=\frac{1}{N}\sum_{i}^{N}\sum_{j}^{N}{\left(x_{i}-y_{j}\right)}^{2}+0.001{\left\|W\right\|}^{2}+Q$$ (1)

where *N* represents the number of ECs, *x*_*i*_ represents the cell-cycle–specific gene expression for an EC from the input layer, *y*_*j*_ represents the cell-cycle–specific gene expression for an EC from the output layer, ||*W*||^2^ represents the regularization of autoencoder weights, and *Q* represents the sparsity parameters (trainAutoencoder, 2021). Visualization and clustering were performed with the UMAP toolkit (McInnes et al., 2018; Meehan, 2021), and clusters of proliferating ECs were defined by co-enrichment for the expression of five proliferation markers (AURKB, MKI67, INCENP, CDCNA8, and BIRC5). We also conducted independent analysis using the entire genome (Supplementary Note S1) and other clustering parameters (Supplementary Note S2) to confirmed that the cell-cycle-specific approach did not miss any specific EC subpopulations.

#### 4.3.2 Trajectory analysis

Cell-cycle–specific trajectory and pseudotime analysis were performed by processing snRNAseq data for the 1,646 cell-cycle–specific genes with the Monocle (v.3) toolkit (Trapnell et al., 2014; Qiu et al., 2017), and pseudotime order was determined by the localization of Cyclin D1, AURKB, and MKI67 expression.

### 4.4 Identification of cluster-specific genes

Upregulated genes for each EC cluster were identified according to the following criteria: 1) a cluster *p*-value (Fisher’s Exact test (Fisher, 1922)) of less than 10^−6^, 2) expression by more than 25% of cells in the cluster, and 3) mean expression at least 2-fold greater than among ECs in other clusters. For transcription factors, which are typically expressed in low abundance, criteria for upregulation were modified to 1) a cluster *p*-value of <10^−6^, 2) expression by >10% of cluster cells, and 3) mean expression >2-fold greater than among cells in other clusters. The list of transcription factors was obtained from the Transcriptional Regulatory Relationships Unraveled by Sentence-based Text mining database (TRRUST) (Han et al., 2018) version 2, and the set of upregulated genes for each cluster was analyzed with the DAVID functional annotation tool (Huang da et al., 2009); only terms present in the Gene Ontology (Huntley et al., 2015) and KEGG (Kanehisa et al., 2017) categories were selected. To avoid false positives caused by multihypothesis testing, the selected results were required to have a Benjamini-adjusted (Li and Barber, 2019) *p* < 0.05.

### 4.5 Immunohistochemistry studies

The immunofluorescence analyses were conducted similar to our previous work in (Nakada et al., 2022). Briefly, samples were cut into transverse sections (thickness: 10 μm), embedded into slides, washed in PBS, and fixed in 4% paraformaldehyde for 20 min at room temperature. Then, the slides were permeabilized at 4°C, 0.1% triton-x for 10 min, and then blocked with Ultra-V block for 7 min. Samples were incubated with mouse anti- PECAM1 at 1:100 (MCA1738, BioRad, United States) or rabbit anti Ki67 (1:100 dilution, ab15580, Abcam, United States), or rabbit antiPDGFRA (1:100 dilution, AF0241, Affinity, United States), or rabbit anti-PLVAP (1:100 dilution, AF0241, Affinity), in PBS containing 10% goat serum (10% PBS)for overnight at 4°C. On the next day, donkey anti-mouse IgG conjugated with FITC at 1:200 dilution and donkey anti-rabbit IgG conjugated with Cy3 at 1; 200 dilution were added for 1 h at room temperature Then, samples were washed after 1:1000 DAPI solution was added for 1 min, mounted in vectorshieldand cover slide for imaging.

The slides were put into an Olympus IX83 microscope to capture images. The image frame size was 1360-width × 1024-height pixels. Exposure time for PECAM1 fluorescence (green) was initially set to 2.8 s, and could be case-by-case adjusted between 2.6 and 3.2 s. Exposure time for Ki67 and PLVAP fluorescence (red) was initially set to 1.2s, and could be adjusted between 0.9 and 1.4 s. Exposure time for PDGFRA fluorescence (red) was always set to 1.2s. DAPI (blue) exposure time was between 12 and 18 μs. Z-stack parameter was set to 0 for all images and channels. For each slide, ten image sets were captured; each image set include three channels: red, green, and blue; then, the three-channels were put together by Matlab to create a ‘merge’ RGB-color image.

Three-channel images (green: PECAM1; red: either Ki67, PDGFRA, or PLVAP; blue: DAPI) were processed using Matlab as in (Nguyen et al., 2022). Multi-thresholding segmentation method (Otsu, 1979) (Matlab multithresh function) was applied to extract image fluorescence regions. The vessel density was computed as the percentage of image area with green fluorescence. EC nuclei were recognized as the DAPI (blue) blocks overlapping with green regions; EC proliferating cells were recognized as the Ki67 red block overlapping with green regions; and EC proliferation marker was computed by the ratio between EC proliferating cells and EC DAPI. EC PDGFRA expression was assessed by the average red intensity overlapping with green fluorescence. For PLVAP + EC analysis, the PECAM1 and PLVAP fluorescence intensity was amplified by 2x before merging into one 3D image channel, PLVAP signal that were not overlapping with PECAM1 was removed for enhanced visualization (PLVAP/DAPI/PECAM1(e) images); then, the number of PLVAP + ECs was manually counted. Statistical comparison among the groups was completed using non-parametric Wilcoxon’s Ranksum test. A *p*-value < 0.05 indicates statistical significance.

## Data Availability

The original contributions presented in the study are included in the article/Supplementary Material; the analytic program is provided at https://github.com/thamnguy/Cardiac-endothelial-cell; further inquiries can be directed to the corresponding author.

## Supplementary material

SUPPLEMENTARY TABLE S1 Summary of upregulated genes, pathways, and biological processes in VEC1. **(A)** Gene expression is summarized as the proportion of VEC1 ECs that express the gene, the fold-change in gene expression relative to expression in non-VEC1 ECs, and the *p*-value (Fisher's Exact test). **(B)** Expression of genes in **(A)** was processed with the DAVID toolkit (https://david.ncifcrf.gov/) to identify pathways and biological processes that were enriched in VEC1; the enrichment *p*-value was corrected to control for false-discoveries.

SUPPLEMENTAL TABLE S2 Summary of upregulated genes, pathways, and biological processes in VEC3. **(A)** Gene expression is summarized as the proportion of VEC3 ECs that express the gene, the fold-change in gene expression relative to expression in non-VEC3 ECs, and the *p*-value (Fisher's Exact test). **(B)** Expression of genes in **(A)** was processed with the DAVID toolkit (https://david.ncifcrf.gov/) to identify pathways and biological processes that were enriched in VEC3; the enrichment *p*-value was corrected to control for false-discoveries.

SUPPLEMENTAL TABLE S3 Sample size *n* (number of pig hearts) per each sample group in our study. The first column indicates the animal group (injury & timepoints), including n. The second column indicates the heart identification. The third and fourth columns indicate whether the heart underwent apical resection on P1 and/or myocardial infarction on P28. The last column indicates the age (day) when the heart tissues were harvested.
