# Supplementary figures and images for: Single-cell RNA sequencing analysis identifies one subpopulation of endothelial cells that proliferates and another that undergoes the endothelial-mesenchymal transition in regenerating pig hearts
Source: Front Bioeng Biotechnol. 2024 Jan 15;11:1257669. doi: 10.3389/fbioe.2023.1257669 (PMC10823534; doi:10.3389/fbioe.2023.1257669)

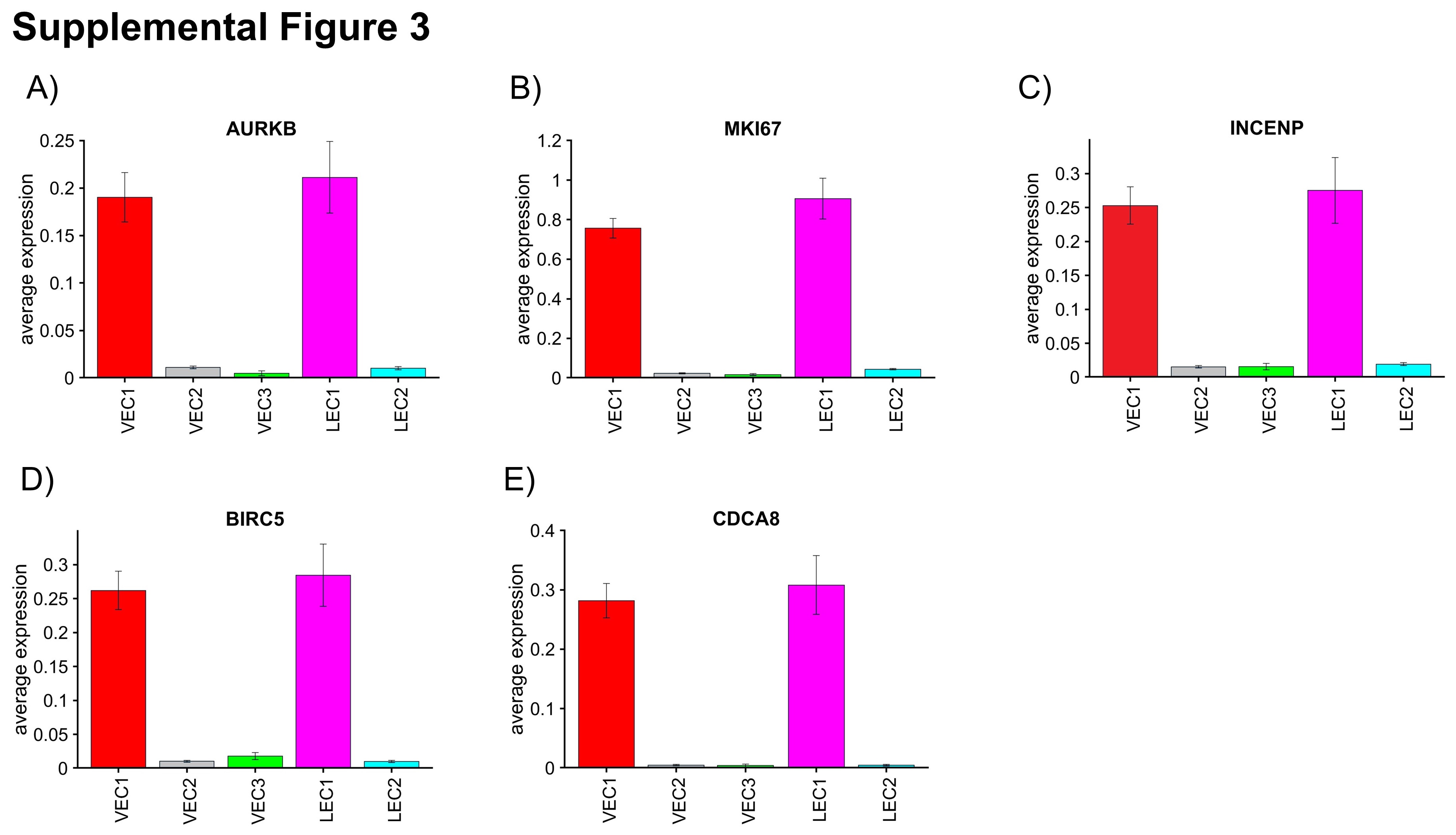

Supplement: Supplementary file 2 [file Image3.jpeg]

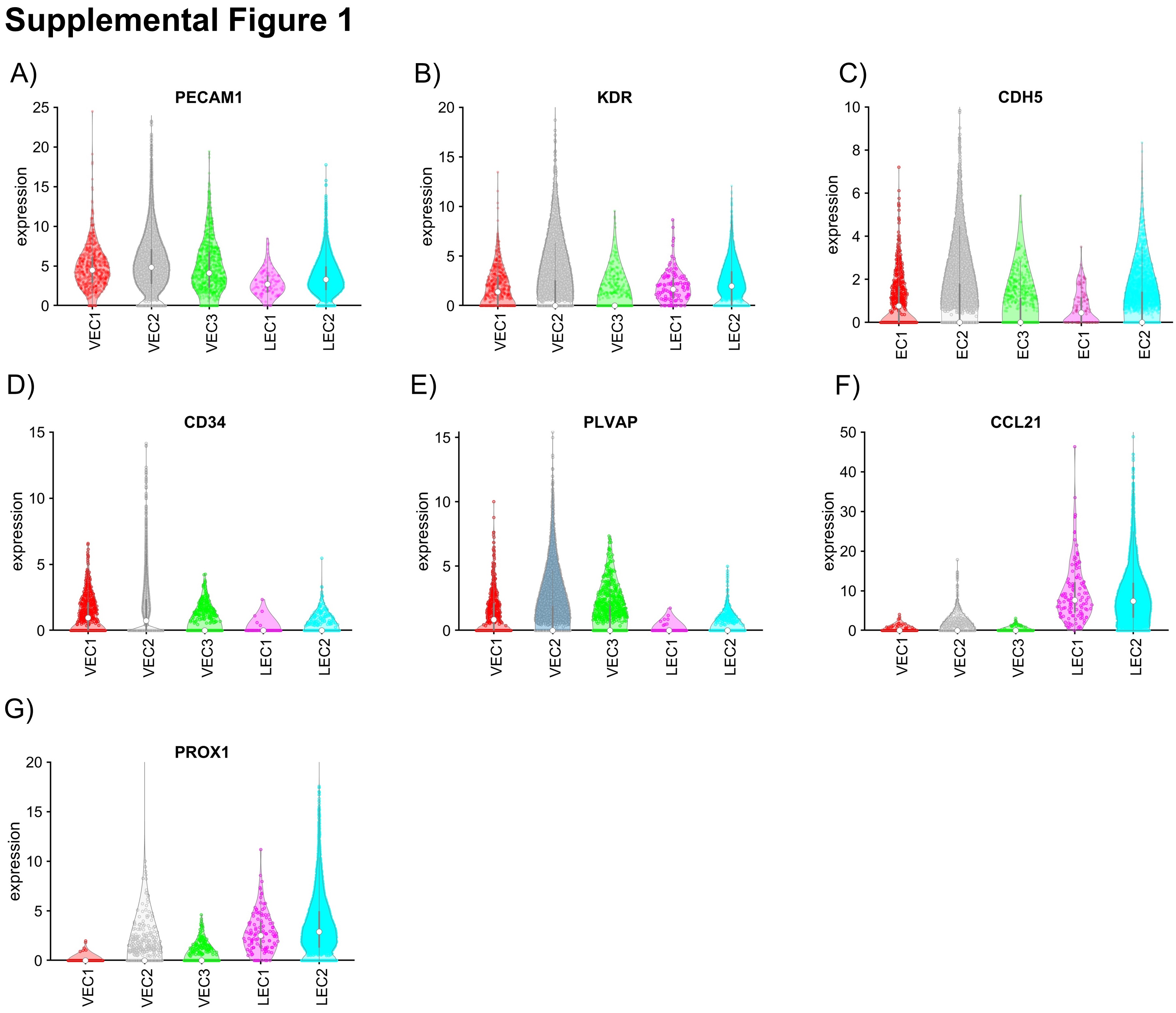

Supplement: Supplementary file 4 [file Image1.jpeg]

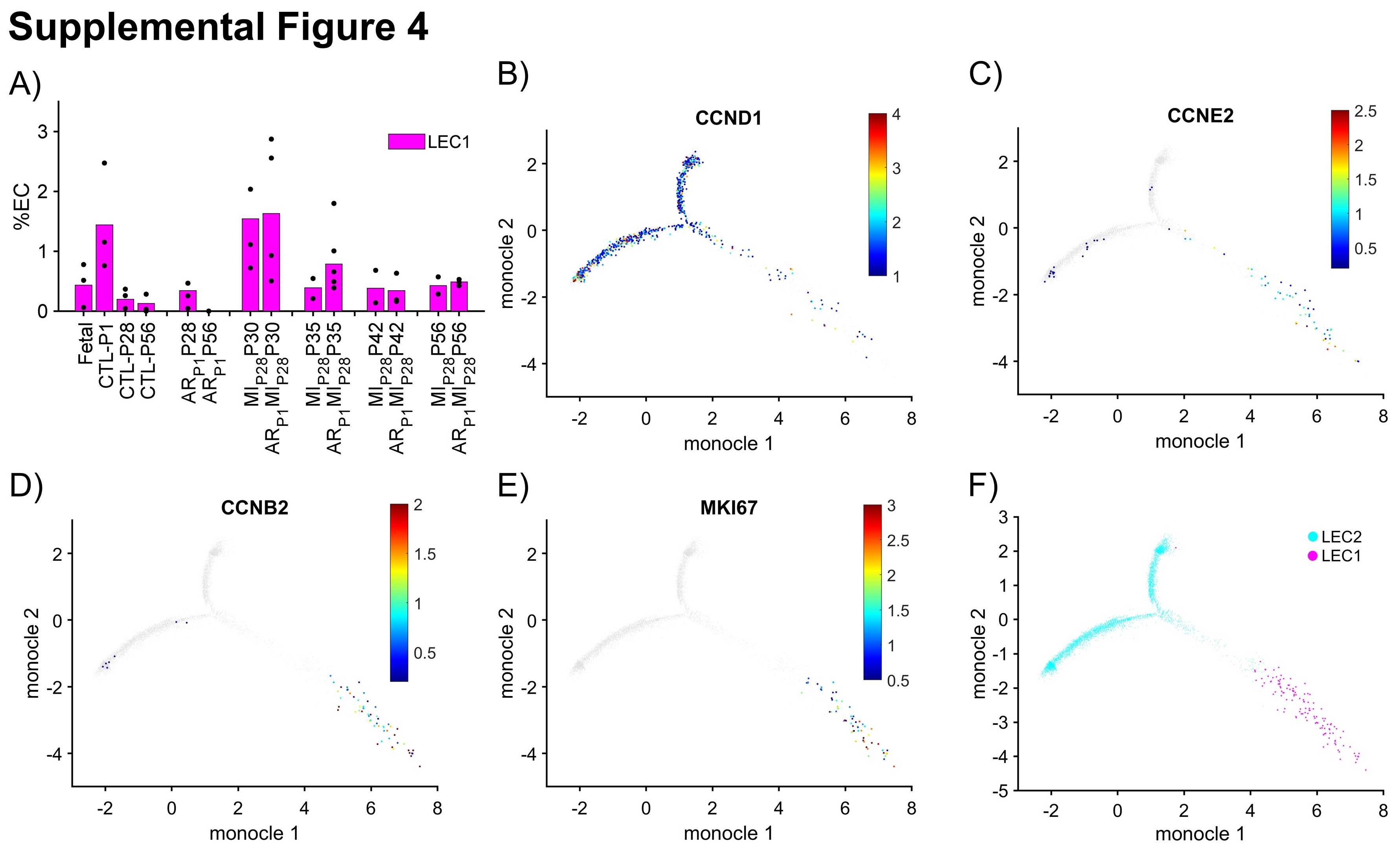

Supplement: Supplementary file 5 [file Image4.jpeg]

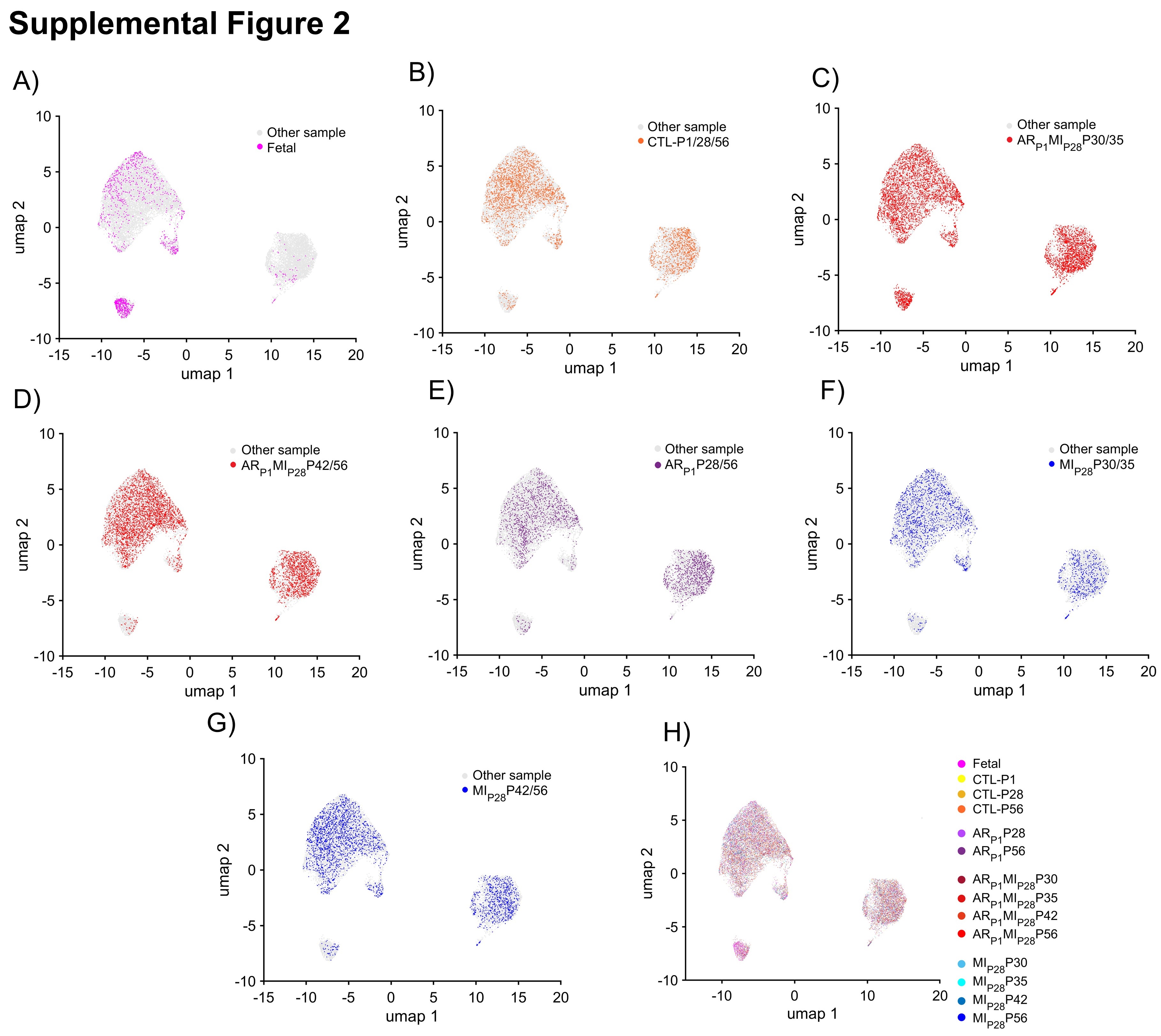

Supplement: Supplementary file 6 [file Image2.jpeg]

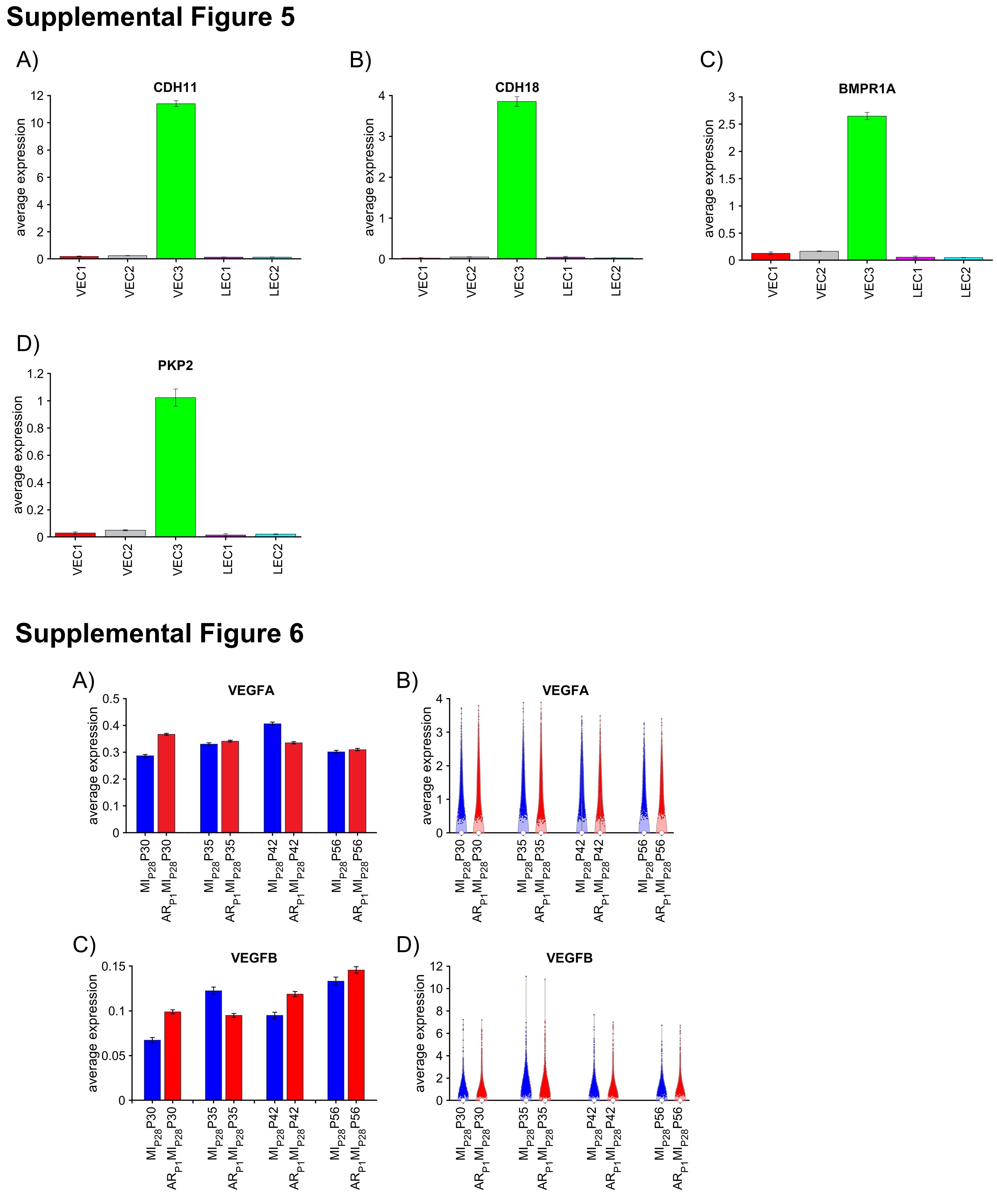

Supplement: Supplementary file 7 [file Image5.jpeg]
