# Supplementary material for: Single-cell RNA sequencing analysis identifies one subpopulation of endothelial cells that proliferates and another that undergoes the endothelial-mesenchymal transition in regenerating pig hearts
Source: Front Bioeng Biotechnol. 2024 Jan 15;11:1257669. doi: 10.3389/fbioe.2023.1257669 (PMC10823534; doi:10.3389/fbioe.2023.1257669)
Supplement: Supplementary file 9 [file DataSheet1.docx]

## Supplemental Note 1. Comparing between Cell-cycle-specific and ‘entire-genome’ snRNAseq analysis

In general, the snRNAseq analysis is conducted using the entire gene list in the genome, which consisted of 25,880 pig genes [1; 2]; on the other hand, in this work, we only used 1646 genes for clustering. Therefore, we independently clustered the EC snRNAseq data using the whole pig genome and compared it with the cell-cycle-specific approach to ensure that the cell-cycle-specific analysis did not lose any specific EC cluster. The entire EC snRNAseq data was embedded by the Autoencoder and clustered using the same parameters to cell-cycle-specific analysis. Then, the cell-cycle-specific clusters (VEC1, VEC2, VEC3, LEC1, and LEC2) were plotted on the UMAP generated by the ‘entire-genome’ analysis. As in Supplemental Note Figure 1, the ‘entire-genome’ analysis only found three major EC clusters; clusters VEC1 and VEC2 were merged into one block, and so did clusters LEC1 and LEC2. Also, the five proliferation markers were spread out, making it difficult to identify cycling cells. Thus, it is clear that compared to the ‘entire -genome’, the cell-cycle-specific analysis did not lose any EC cluster; furthermore, the cell-cycle-specific was superior in identifying cycling EC, which was the main finding in angiogenesis.

## Supplemental Note 2. Examining clustering parameters in Cell-cycle-specific analysis

In snRNAseq analysis, using different clustering parameters may result in different numbers of clusters. As the number of clusters increases, small clusters having too few number of cluster-specific markers would appear; seeing this case, we did not further change clustering parameters. For example, in Supplemental Note Figure 2, we attempted a different clustering parameter in the cell-cycle-specific analysis, in which a small cluster, called VEC*, was defined, and this cluster appeared to be transiting toward the cycling VEC1 cluster. However, analyzing this cluster’s specific markers only yielded five genes ND2, COX1, COX2, ATP6, ND4, and CYTB. Therefore, these VEC* cells appeared to have no important biological interpretation; it indicated that we should not further increase the number of clusters.

## Supplemental Note Figures


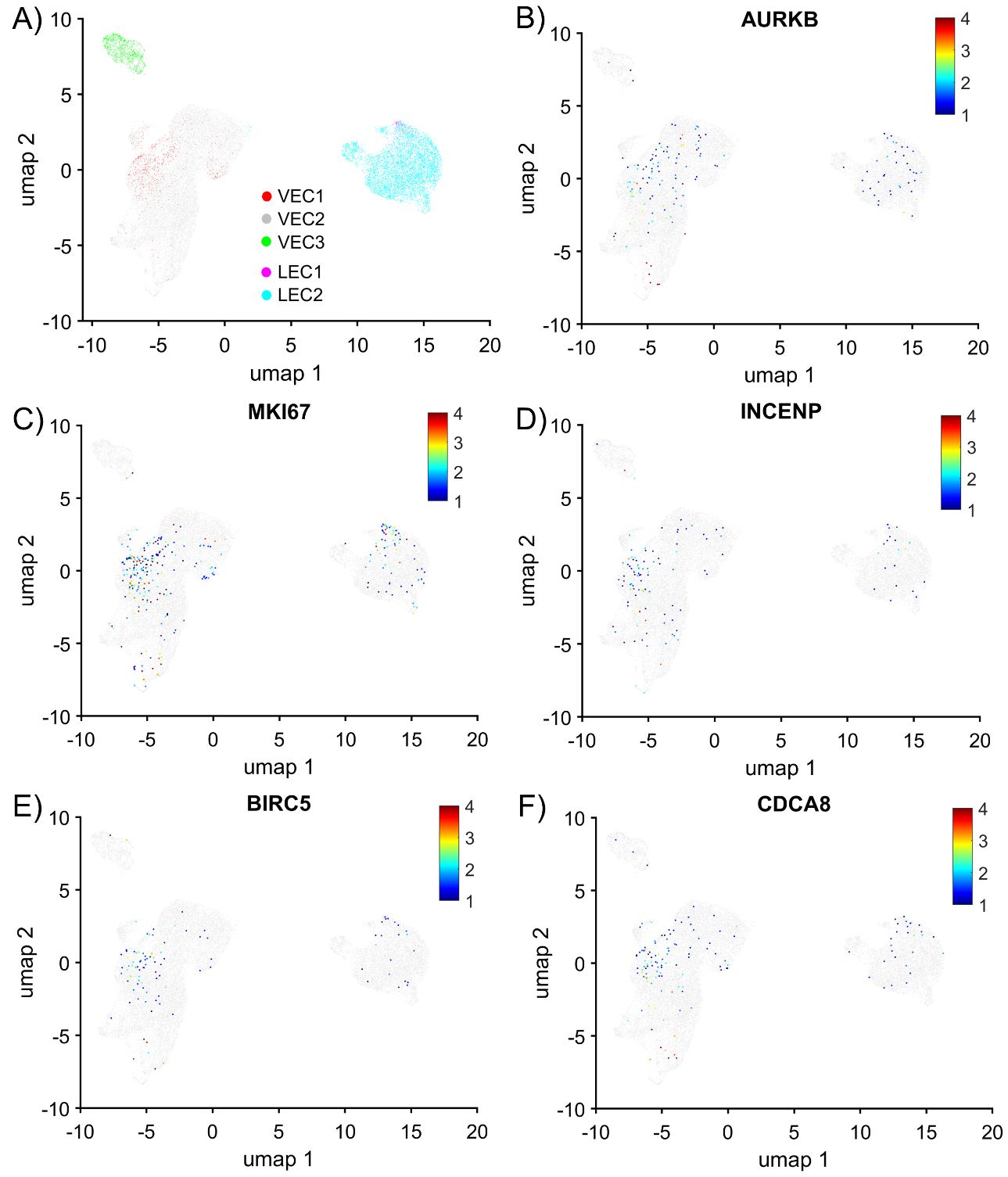


**Supplemental Note Figure 1**. Analyzing and visualizing EC using the ‘entire-genome’ analysis. The EC snRNAseq data was embedded by an ‘entire-genome’ autoencoder and projected onto a 2D UMAP, using the same algorithm parameters to the cell-cycle-specific analysis. (A) entire-genome UMAP plot, the cells were labeled using the cell-cycle-specific cluster results (five clusters VEC1-3 and LEC1-2). (B-F) Cells that express the mitosis and cytokinesis markers (B) AURKB, (C) MKI67, (D) INCENP, (E) BIRC5, and (F) CDCA8 are displayed across the 2D ‘entire-genome’ UMAP with the magnitude of expression represented as a color-coded heatmap; gray ECs did not express the corresponding molecule.


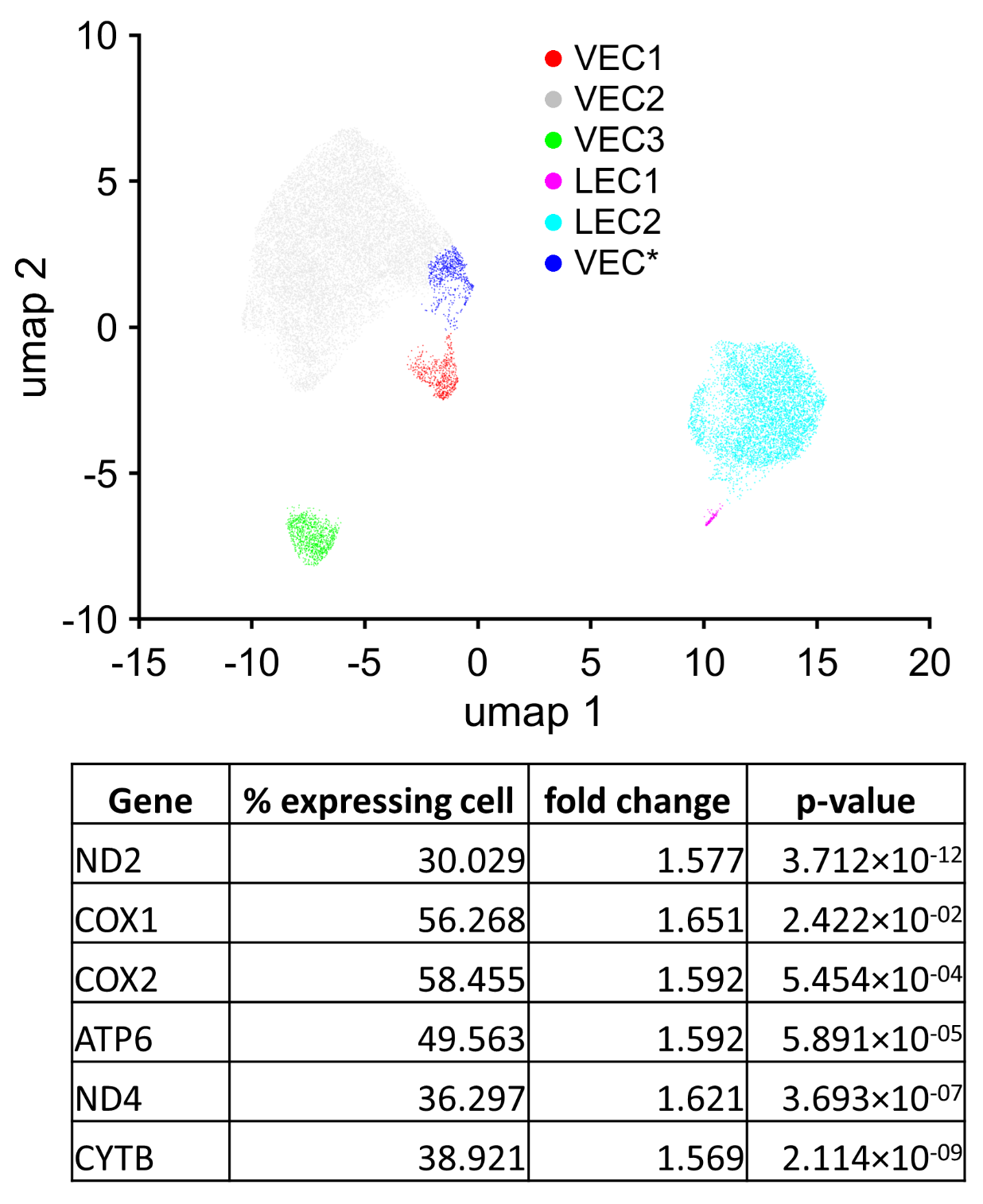


**Supplemental Figure Note 2**. Attempting to cluster the EC using a different clustering parameter, yielding a VEC* cluster, which appears to be neighbor to cluster VEC1. (Top) Localization of VEC* and other clusters, computed by the cell-cycle-specific UMAP. (Bottom) Statistical table of marker genes in VEC*; table header includes: the proportion of VEC* cells that express the gene, the fold-change in gene expression relative to expression in VEC*, and the p-value (Fisher's Exact test)

## References

[1] T. Nguyen, Y. Wei, Y. Nakada, J.Y. Chen, Y. Zhou, G. Walcott, and J. Zhang, Analysis of Cardiac Single-cell RNA-sequencing Data Can be Improved by the Use of Artificial-Intelligence-based Tools. Scientific Reports 13 (2023) 6821.

[2] T. Nguyen, Y. Wei, Y. Nakada, Y. Zhou, and J. Zhang, Cardiomyocyte Cell-Cycle Regulation in Neonatal Large Mammals: Single Nucleus RNA-Sequencing Data Analysis via an Artificial-Intelligence-Based Pipeline. Front Bioeng Biotechnol 10 (2022) 914450.
